# Concurrent Obsessive-Compulsive Symptoms in Patients With Schizophrenia: A Retrospective Study From a Tertiary Care Centre in Sindh, Pakistan

**DOI:** 10.7759/cureus.37583

**Published:** 2023-04-14

**Authors:** Marium Shoaib, Maria Iqbal, Uzma J Waqas, Sheikh M Ahmed, Fnu Sangeet, Fatima A Raza, Azka Shahab, Kiran Fatima, Maham Siddiqui, Ammar Nadeem

**Affiliations:** 1 Department of Acute Medicine, Blackpool Victoria Hospital, Lancashire, GBR; 2 Department of Surgery, Blackpool Victoria Hospital, Lancashire, GBR; 3 Department of Medicine, Jinnah Postgraduate Medical Centre, Karachi, PAK; 4 Department of Medicine, Liaquat National Hospital, Karachi, PAK; 5 Department of Psychiatry, Jinnah Postgraduate Medical Centre, Karachi, PAK; 6 Department of Medicine, Karachi Medical and Dental College, Karachi, PAK; 7 Department of Medicine, Pakistan Navy Station Shifa Hospital, Karachi, PAK; 8 Department of Medicine, Jinnah Sindh Medical University, Karachi, PAK

**Keywords:** schizophrenic, schizoprenia, schizo-obsessive disorder, psychiatric co-morbidity, obsessive compulsive disorders

## Abstract

Introduction: The present study aimed to evaluate the proportion of concurrent symptoms of obsessive-compulsive symptoms (OCSs) among patients with schizophrenia.

Methods: A retrospective study was undertaken at the Department of Psychiatry, Jinnah Postgraduate Medical Center, Sindh, Pakistan between 1st March 2019 and 1st April 2020. All cases with diagnosed schizophrenia irrespective of gender, age, or ethnicity were eligible for the study. We excluded patients with acute psychosis due to isolated substance use disorder or any organic brain disease. The medical records for each patient were retrieved from the departmental database. Sociodemographic factors including age, gender, ethnicity, and presence of OCSs and other psychiatric comorbidities were recorded in a predefined pro forma. The presence of OCSs was noted by the attending psychiatrist during history taking as positive or negative.

Results: A total of 139 patients were included. A predominance of the male gender was noted. There were 63 (45.3%) patients with concurrent OCSs. Out of the total patients, 42 (66.67%) males and 21 (33.33%) females had OCSs. A total of 28 (44.44%) patients between 31 and 45 years of age had OCSs. Out of the 63 patients with OCSs, 36 (57.14%) had a history of substance abuse (p = 0.471). In the study, 17 (26.98%) Balochi and 19 (30.16%) Pashtuns had OCSs. However, the difference was statistically insignificant.

Conclusion: In conclusion, OCSs were frequent in patients with schizophrenia, according to the current study. We discovered that males, individuals between the ages of 18 and 30 years, Balochis, Pashtuns, and those with a history of substance abuse were more likely to have OCSs. However, the difference was not statistically significant.

## Introduction

Schizophrenia is a psychiatric disease that affects cognition, emotion, perception, and other aspects of an individual's behavior causing disruption in the day-to-day activities of a patient [1]. Schizophrenia is among the most common causes of disability globally, affecting an estimated population of 1% [2].

The burden of schizophrenia was approximately 0.28% in 2016 irrespective of gender. Globally, the number of cases rose from 13.1 million to 20.9 million cases within two decades [2]. Schizophrenia is associated with 13 million years of life lived with disability highlighting the substantial burden of the disease. The proportion of schizophrenia cases has risen at an unsettling rate in Pakistan, as compared to other mental diseases [3]. A study from Pakistan revealed that Schizophrenia was the most common disorder among both males (30.4%) and females (25.2%) in the overall sample [4].

In the last few decades, literature has emerged highlighting the co-existence of psychiatric disorders in individuals with schizophrenia. Comorbid conditions in schizophrenia, including obsessive-compulsive symptoms (OCSs), impaired cognition, depression, anxiety, and substance abuse, have an impact on the management plan, patient compliance to medication, and patient outcomes [4-6]. The occurrence of OCSs in individuals with schizophrenia has been disclosed in recent studies with a varying proportion from 10% to 64% [5-7].

Preliminary studies demonstrated a positive outcome and suggested the relationship between OCSs and schizophrenia was an uncommon phenomenon. However, more recent research has shown that OCSs are present in a considerably larger proportion of schizophrenia patients and have a negative impact on the course and severity of the illness [4-6].

According to a meta-analysis of 50 studies published in 2011, 38.3% of individuals with schizophrenia had anxiety issues [7]. Obsessive-compulsive disorder (OCD) was reported in 12.1% of patients with schizophrenia, which is a higher incidence than in the general population [8]. These findings were supported by another meta-analysis involving 3978 patients with schizophrenia, with a prevalence of OCD of 12.3% and OCS of 30.3% [9].

Schizophrenia is a complex mental disorder that has a wide array of symptoms. It is often difficult to diagnose because its symptoms can be similar to those of other mental health conditions, such as OCD, bipolar disorder, or major depressive disorder. Comorbid psychiatric illnesses are harder to diagnose and treat. Furthermore, many people with schizophrenia may not seek treatment due to stigma or a lack of understanding about their condition [10]. Thus, it would be useful for psychiatrists to understand the symptomatology of schizophrenia and its association with comorbid psychiatric illnesses. The present study aimed to highlight the burden of comorbid OCSs in patients with schizophrenia. The findings of the study would identify the need for individualized treatment plans for better patient outcomes.

## Materials and methods

A retrospective study was undertaken at the Department of Psychiatry, Jinnah Postgraduate Medical Center, Sindh, Pakistan between 1st March 2019 and 1st April 2020. The data acquisition was started after obtaining permission from the Institutional Review Board (IRB) of Jinnah Postgraduate Medical Center with the reference number 2019-GENL/7678.

All cases of diagnosed schizophrenia presented between 1st January 2015 and 28th February 2019 were eligible to be entered in the study. All cases with diagnosed schizophrenia, irrespective of gender, age, or ethnicity, were eligible for the study. We excluded patients with acute psychosis due to isolated substance use disorder or any organic brain disease.

The OpenEpi software (https://www.openepi.com/SampleSize/SSMean.htm) was used to determine the required sample size. By keeping the estimated prevalence of OCSs among patients with schizophrenia at 10% [11], a margin of error at 5%, and a confidence level of 95%, a sample size of 139 was determined.

Since this was a retrospective study, informed written consent of the patient was not applicable. The medical records for each patient were retrieved from the departmental database from 1st March 2018 to 30th March 2019. All cases of schizophrenia were diagnosed by an experienced consultant psychiatrist. As per the Diagnostic and Statistical Manual of Mental Disorders (DSM-5), an individual was diagnosed with schizophrenia if at least two of these five main symptoms (delusions, hallucinations, speaking incoherence, unusual movements, and negative symptoms) were present for at least six months and have significantly disrupted the individual’s ability to work or maintain relationships [12]. Furthermore, the presence of either obsessive or compulsive symptoms or both were identified using an operational definition. Obsessive symptoms were defined as persistent, repetitive, intrusive, and distressful thoughts unrelated to the individuals’ delusions while the compulsions were defined as repetitive goal-directed rituals clinically different from mannerisms of schizophrenia [13]. Presence of OCSs was noted by the attending psychiatrist during history taking as positive or negative.

Substance use disorders were defined as uncontrollable use of alcohol and/or drugs that has significant effects on the users' health and their ability to function and meet responsibilities at work, school, or home. The DSM-5 criteria for diagnosing a substance use disorder include signs of impaired control, social problems, risky use, and specific pharmacological criteria [12].

The medical records of all eligible cases were recruited for data extraction. Any personal identifiers such as names, hospital numbers, or home addresses were not collected to maintain the anonymity of the patients. Sociodemographic factors including age, gender, ethnicity, and presence of OCSs and other psychiatric comorbidities were recorded in a predefined pro forma.

Cases with partial or incomplete history or incomplete note-taking at admission or presentation were not included in the final analysis. Data was entered into IBM SPSS Statistics for Windows (Version 23.0. Armonk, NY: IBM Corp.). All continuous data such as age were illustrated as an average. All categorical data including gender, presence of OCSs, and substance use disorder were presented as frequency and proportions. The chi-square test was used to find the impact of sociodemographic and clinical parameters on the concurrence of OCSs in patients with schizophrenia. A p-value of ≤ 0.05 was deemed statistically significant.

## Results

A total of 139 patients with a mean age of 31.50 ± 9.39 years were included in the study. Of these, 63 (61.17%) were males and 40 (38.83%) were females. There were 66 (47.48%) individuals between the age of 16 and 30 years while 73 (52.52%) were 30 years or older. There was a preponderance observed toward Balochi and Pashtuns with a proportion of 40 (28.78%) and 39 (28.06%), respectively. A total of 75 (53.9%) patients had a history of substance abuse (Table 1).

**Table 1:** Sociodemographic profile of patients with schizophrenia (n = 139)

Parameters	N (%)
Gender	
Male	88 (63.31%)
Female	51 (36.69%)
Age	
16-30 years	66 (47.48%)
31-45 years	73 (52.52%)
Ethnicity	
Urdu speaking	22 (15.83%)
Sindhi	22 (15.83%)
Punjabi	16 (11.51%)
Balochi	40 (28.78%)
Pashtun	39 (28.06%)
Substance abuse	
Yes	75 (53.9%)
No	64 (46.0%)

There were 63 (45.3%) patients with concurrent OCSs. A total of 42 (66.67%) males and 21 (33.33%) females had OCSs. There were 28 (44.44%) patients between 31 and 45 years of age who had OCSs. Out of the 63 patients with OCSs, 36 (57.14%) also suffered from substance abuse (p = 0.471). In the study, 17 (26.98%) Balochi and 19 (30.16%) Pashtuns had OCSs, albeit the difference was not statistically significant (Table 2). Out of the 75 patients with a history of a substance use disorder, 61 (81.3%) used Cannabis (bhang/chars), eight (10.7%) used N-methyl-D-aspartate (NMDA), three (4%) used phencyclidine, and three (4%) used Naswar. 

**Table 2:** Association of sociodemographic parameters with OCSs among patients with schizophrenia (n = 139) OCSs, obsessive-compulsive symptoms

Parameters	Presence of OCSs	Absence of OCSs	P-value
Gender			0.365
Male	42 (66.67%)	45 (59.21%)	
Female	21 (33.33%)	31 (40.79%)	
Age			0.419
16-30 years	28 (44.44%)	39 (51.32%)	
31-45 years	35 (55.56%)	37 (48.68%)	
Ethnicity			0.892
Urdu speaking	11 (17.46%)	10 (13.16%)	
Sindhi	9 (14.29%)	13 (17.11%)	
Punjabi	7 (11.11%)	10 (13.16%)	
Balochi	17 (26.98%)	24 (31.58%)	
Pashtun	19 (30.16%)	19 (25.00%)	
Substance abuse			0.471
Yes	36 (57.14%)	39 (51.32%)	
No	27 (42.86%)	37 (48.68%)	

## Discussion

The present study retrospectively assessed the medical records of individuals with schizophrenia for the presence of OCSs. Furthermore, the impact of age, gender, ethnicity, and substance abuse was sought. The current study divulged that 45.3% of individuals had concurrent OCSs. The majority of the individuals with schizophrenia belonged to Balochi and Pashtun ethnicity. There was also a male preponderance observed in the study. Furthermore, the substance abuse rate was alarmingly high among the patients. Age, gender, ethnicity, and substance abuse did not differ significantly. However, we could not find any statistical difference between OCSs.

The current study findings were in accordance with published literature. A study published by Kontis et al. studied 110 patients with schizophrenia and assessed OCSs among them. The study revealed that 51 patients had at least one OCS. Interestingly, the study further noted that patients with at least one OCS had better social functioning than those without OCS [14].

Another study supported this finding revealing that patients with schizophrenia have a U-shaped relationship between functioning and the presence of OCSs. The study concluded that mild OCSs had a direct association with better functioning, whereas moderate and severe OCSs had an inverse relationship with functioning [15].

In contrast to our study, Ahn Robins et al. assessed about 22 thousand patients with schizophrenia and other psychotic disorders. Out of these, 24% had OCSs and 11.9% had OCD. Although the rate of OCSs is much lower compared to our study, the study revealed that individuals with either OCSs or OCD had an increased likelihood of aggressiveness (odds ratio = 1.18; 95% CI, 1.10-1.26) and cognitive impairment (odds ratio = 1.21; 95% CI, 1.13-1.30) [16].

The concurrence of OCSs and OCD has an impact on the severity of schizophrenia, and it influences the disease course and treatment plans. A meta-analysis concluded that individuals with schizophrenia and concurrent OCS or OCD had increased severity of psychotic symptoms (p = 0.0104) [17]. There are certain studies claiming that certain antipsychotics exacerbates the OCSs severity among patients with schizophrenia [18-20].

Since this was a retrospective study, it was riddled with several limitations. First, due to the convenience sampling technique, the data could not fairly represent the general population and was prone to selection bias. Furthermore, we could not establish a causation relationship of schizophrenia with OCSs. Thus, we recommend that further multi-center studies should be undertaken to overcome the above-mentioned limitations.

## Conclusions

The present study revealed that OCSs were highly prevalent in patients with schizophrenia. We found a preponderance of OCSs in males, individuals aged between 18 and 30 years, Balochi, Pashtuns, and those who had a history of substance abuse. However, the study did not find statistically significant evidence of an association between age, gender, ethnicity, and substance abuse with OCSs.
